# Supplementary material for: The Study of Cerebrospinal Fluid microRNAs in Spinal Cord Injury and Neurodegenerative Diseases: Methodological Problems and Possible Solutions
Source: Int J Mol Sci. 2021 Dec 22;23(1):114. doi: 10.3390/ijms23010114 (PMC8744986; doi:10.3390/ijms23010114)
Supplement: Supplementary file 1 [file ijms-23-00114-s001.zip › ijms-1508933-supplementary.pdf]

Table S1. miRNA expression in spinal cord and cerebrospinal fluid at different periods post-injury.

Organism / Type of SCI	*miRNA*	*Expression changes/Period post-SCI*	*Targets*	*Potential effects*	*References*
SPINAL CORD					
Adult female SD rats / T10 contusion	miR-137	↓ 7 days	Caspase 3; Calpain2	pro-apoptotic role	[16]
	miR-235-3p		Caspase 3		
	miR-124		Calpain 1		
	miR-30b-3p		AIF		
	miR-99		Calpain 3		
	miR-181a		cPLA2	inflammatory related	
	miR-127		sPLA2		
	miR-411		TNFα		
	miR-99a		TNFα; ICAM1		
	miR-30c, miR-30b-5p, miR-384-5p		IL-1β		
	miR-34a		ICAM		
	miR-145, miR-214, miR-133a	↑ 7 days	Bcl2-1	anti-apoptotic role	
	miR-672, miR-103, miR-206, miR-133b		Bcl2-2		
	miR-107, miR-20a, miR-17, miR-674-5p				
	miR-152		Catalase; Bcl2-1		
	miR-221		ANXA1	anti-inflammatory related	
	miR-1, miR-206		ANXA2, SOD1	anti-oxidative factor	
	miR-214		Catalase	anti-inflammatory related or anti-oxidative factor	
Adult male SD rats / T12-T13 contusion	miR-1	↓ 4, 14 days	N/A	cell cycle	[27]
	miR-124		SOX2	differentiation of cells	
	miR-129-1	↓ 14 days	CDK-6	cell cycle entry could ultimately lead to significant neuronal death	
	miR-129-2		CDK-7		
	miR-146a	↑ 4 days	IL-6;IL- 8;IL-1β;TNFα	anti-inflammatory related and promotion of cell proliferation	
	miR-21	↑ 4, 14 days	nestin	anti-apoptotic role	
	miR-223	↑ 14 days	N/A	inflammation related	

Adult female Wistar rats / T8 contusion	miR-29b	↓ 3 days	MCL1 activate p53 pathway	pro-apoptotic role	[11]
	miR-7		EGFR-regulating AKT pathway		
	miR-1	↓ 3, 7 days	BCL-2, HSP60, HSP70, IGF1		
	miR-34		SIRT1, BCL-2		
	miR-148b		PRKCZ		
	miR-138		BCL-2		
	miR-let-7g		cdc42, SOX3	inflammatory related	
	miR-124		In quiescent microglia	associated with immune cells	
	miR-9		NF-κB	anti-inflammatory related	
	miR-133b		PTPPF, CASP-9, BCL-W, MCL-1	pro-apoptotic and anti-apoptotic role	
	miR-341		PTPRF	cell death	
	miR-125b	↓ 7 days	TNF-α	anti-inflammatory related	
	miR-101		MCL-1	pro-apoptotic role	
	miR-10		BIM	anti-apoptotic role	
	miR-96		FOXO1, CASP-3, FADD		
	miR-223	↑ 1, 3, 7 days	IKK α	associated with immune cells and anti-inflammatory related	
	miR-142		T-cells	associated with immune cells	
	miR-15b,	↑ 3 days	BCL-2	pro-apoptotic role	
	miR-17-92		BIM, E2F1, PTEN, TGFBR2, PSMAP2, SMAD4	anti-apoptotic role and pro-inflammatory related	
	hsa-miR-106a		IL10	pro-inflammatory related	
	miR-21	↑ 3, 7 days	TPM, PTEN, PDCD4	anti-apoptotic role, pro and anti-inflammatory related	
	miR-146a	↑ 7 days	TRAF-6, IRAK1, FAS	anti-apoptotic role and anti-inflammatory related	
Adult female ICR mice / T11 contusion	miR-486	↑ 7 days	NeuroD6	neuroprotective role against reactive oxygen species-mediated apoptotic neuronal cell death	[81]
Adult female ICR mice / T9,T10 transection	miR-125b-3p, miR-128a-pre, miR-137 miR-129-2-pre, miR-136, miR-135a,b	↓ 2 days	N/A	N/A	[82]
	miR-20a	↑ 2, 7 days	Ngn1, Ngn2	motor neuron and myelin destruction	
Adult female SD rats / T10 transection	miR-21	↑ 10 days	PTEN	regeneration of corticospinal tract axons	[83]
	miR-199a-3p	↑ 10, 31 days	mTOR	factor in an intracellular signaling pathway that regulates protein synthesis, cell growth and proliferation	

Adult male SD Rats / T10 contusion	miR-708, miR-125-3p	↓ 1 day	N/A	N/A	[84]
	miR-let-7b, miR-210, miR-126, miR-24	↓ 1, 3 days			
	miR-410, miR-27b,miR-99a	↓ 3 days			
	miR-21	↑ 1, 3 days	FaSL, PTEN, PDCD4	anti-apoptotic factor	
	miR-760-5p, miR-463, miR-473b, miR-291b, miR-336, miR-425, miR-134, miR-1188-3p, miR-539	↑ 1 day	N/A	N/A	
	miR-3591, miR-17-5p	↑ 3 days			
Adult female C57BL/6 mice / T10 contusion	miR-9, miR-219, miR-384-5p	↑ 12 hours	N/A	oligodendrocyte differentiation and myelin maintenance	[85]
BLOOD SERUM					
Adult C57BL/6 female mice / T10 contusion	miR-1285	↓ 1, 3, 5 days	N/A	N/A	[85]
	miR-4331	↓ 1, 3 days			
	miR-146a-5p,miR-155	↑ 3, 12 hours			
	miR-9			oligodendrocyte differentiation and myelin maintenance	
	miR-219, miR-384-5p	↑ 12 hours			
	miR-1, miR-125b	↑ 1 day		N/A	
	miR-133b, miR-208b, miR-27b-3p, miR-133a-3p	↑ 1, 3 days			
	miR-128, miR-130a, miR-378b-3p miR-133a-5p, miR-22-5p	↑ 1, 3, 5 days			
	miR-let-7a, miR-let-7g, miR-301	↑ 3, 5 days			
Adult SD female rats / T9 contusion	rno-miR-125b-5p	↓ 6 hours	N/A	negative regulator of p53 and p53-induced apoptosis	[86]
	novel_mir-803, novel_mir-513, novel_mir-2364			N/A	
	novel_mir-1945, novel_mir-1985, novel_mir-429				
	novel_mir-154, novel_mir-4,				
Adult SD female rats / T9 contusion	rno-miR-152-3p	↑ 6 hours	N/A	negative regulator of innate immune response and antigen presentation	[86]
	rno-miR-130a-3p			substance P synthesis and release of mesenchymal stem cells (MSC)-derived neuronal cells	
	novel_mir-2064, rno-miR-126a-5p, novel_mir-39			N/A	
	rno-miR-466b-5p, rno-miR-1188-5p				
	novel_mir-603, novel_mir-51, novel_mir-1387				

Human / AIS A, B, C	miR-208, miR-499	↑ 1 day	N/A	Regulators of skeletal muscle mass	[18]
	miR-133a-3p			N/A	
CSF					
Human / AIS A, B, C	miR-23b-3p, miR-195-5p	↓ 1 day	N/A	N/A	[18]
	miR-9, miR-219, miR-10b	↑ 1 day			
	miR-21		Faslg	cell death and attenuation of the hypertrophic response to SCI	

SD – Sprague-Dawley rats; N/A – not available; AIS – American Spinal Injury Association (ASIA) Impairment Scale; SCI – spinal cord injury; CSF – cerebrospinal fluid.
